# HuR-targeted small molecule inhibitor exhibits cytotoxicity towards human lung cancer cells

**DOI:** 10.1038/s41598-017-07787-4

**Published:** 2017-08-30

**Authors:** Ranganayaki Muralidharan, Meghna Mehta, Rebaz Ahmed, Sudeshna Roy, Liang Xu, Jeffrey Aubé, Allshine Chen, Yan Daniel Zhao, Terence Herman, Rajagopal Ramesh, Anupama Munshi

**Affiliations:** 1 Department of Pathology, University of Oklahoma Health Sciences Center, Oklahoma City, Oklahoma 73104 USA; 2 Department of Radiation Oncology, University of Oklahoma Health Sciences Center, Oklahoma City, Oklahoma 73104 USA; 3 Department of Biostatistics and Epidemiology, University of Oklahoma Health Sciences Center, Oklahoma City, Oklahoma 73104 USA; 4 Stephenson Cancer Center, University of Oklahoma Health Sciences Center, Oklahoma City, Oklahoma 73104 USA; 5 Graduate Program in Biomedical Sciences, University of Oklahoma Health Sciences Center, Oklahoma City, Oklahoma 73104 USA; 6 Division of Chemical Biology, University of North Carolina, Chapel Hill, North Carolina 27599 USA; 7 Department of Molecular Biosciences, University of Kansas Medical Center, Kansas City, 66160 Kansas USA

## Abstract

Human antigen (Hu) R is an RNA-binding protein whose overexpression in human cancer correlates with aggressive disease, drug resistance, and poor prognosis. HuR inhibition has profound anticancer activity. Pharmacologic inhibitors can overcome the limitations of genetic inhibition. In this study, we examined the antitumor activity of CMLD-2, a small-molecule inhibitor directed against HuR, using non-small cell lung cancer (NSCLC) as a model. CMLD-2 efficacy was tested *in vitro* using H1299, A549, HCC827, and H1975 NSCLC cells and MRC-9 and CCD-16 normal human fibroblasts. Treatment of NSCLC cells with CMLD-2 produced dose-dependent cytotoxicity, caused a G1 phase cell-cycle arrest and induced apoptosis. CMLD-2 decreased HuR mRNA and the mRNAs of HuR-regulated proteins (Bcl2 and p27) in tumor cells. Additionally, reduction in the expression of HuR, Bcl2, cyclin E, and Bcl-XL with increased expression of Bax and p27 in CMLD-2-treated NSCLC cells were observed. CMLD-2-treated normal cells, HuR-regulated mRNAs and proteins albeit showed some reduction were less compared to tumor cells. Finally, CMLD-2 treatment resulted in greater mitochondrial perturbation, activation of caspase-9 and -3 and cleavage of PARP in tumor cells compared to normal cells. Our proof-of concept study results demonstrate CMLD-2 represents a promising HuR-targeted therapeutic class that with further development could lead to advanced preclinical studied and ultimately for lung cancer treatment.

## Introduction

HuR is an RNA-binding protein that regulates the stability and transcription of numerous mRNAs whose protein products function as oncoproteins and are frequently overexpressed in several human cancers, including lung cancer^1–3^. HuR overexpression has been correlated with aggressive disease and poor prognosis^4–10^. Preclinical studies have demonstrated that HuR promotes tumor cell proliferation, migration, angiogenesis, and metastasis^11–14^. Further, HuR overexpression has been reported to contribute to drug resistance^15–17^. Results from these preclinical and clinical studies suggest that HuR may be a molecular target for cancer therapy and that suppression of HuR will likely result in tumor growth inhibition and anticancer activity.

Studies from our laboratory and others have previously shown that inhibition of HuR expression by gene silencing inhibited cell proliferation, migration, invasion, angiogenesis, and metastasis in a broad spectrum of human cancer cells^11–14, 18–22^. These studies utilized anti-sense oligonucleotide or small interfering (si) RNA to inhibit HuR. While these results established proof-of-concept, there are several barriers, such as poor cell uptake and low serum stability, to siRNA-based therapy. Another challenge is the availability of a delivery vehicle that can efficiently deliver the HuR-targeted si/shRNA, oligonucleotide, or plasmid DNA to tumor depots and produce considerable anticancer activity. While several formulations for siRNA delivery have been developed and tested, each of the formulations has its limitations^23–26^. Thus, approaches that utilize genetic inhibition for cancer treatment often suffer from issues related to inefficient drug delivery to tumor tissues, thus limiting their clinical translation.

More recently, we developed and tested tumor-targeted nanoparticle delivery of HuRsiRNA (HuR-NP) in lung cancer, and showed significant antitumor activity *in vitro* and *in vivo*. The combination of HuR-NP with a CXCR4 inhibitor produced enhanced inhibition of lung tumor cell migration and invasion^26^. Similarly, HuR siRNA therapy combined with ionizing radiation resulted in significant radiosensitization of triple-negative breast cancer (TNBC) cells^27^. Consistent with our results, other laboratories have reported similar treatment outcomes with HuR siRNA^11–14, 28^. While all of these studies are promising, advancing siRNA-based therapy to the clinic remains a challenge. The availability of a pharmacologic inhibitor directed against HuR, however, offers advantages over siRNA based therapies and can be rapidly tested and advanced for clinical studies. Blanco *et al*.^29^, using MS-444, an HuR-targeted inhibitor, showed antitumor activity against colorectal cancer both *in vitro* and *in vivo*. Concurring with these findings, Romeo *et al*.^30^, showed MS-444 treatment reverted TRAIL resistance in pancreatic cancer. Both studies provide evidence that small molecule inhibitors can successfully disrupt HuR and produce anticancer activity. More recently, Wu *et al*.^31^, reported the development of a small molecule inhibitor (CMLD-2) that disrupts the interaction between HuR protein and its target mRNA.

CMLD-2 is a coumarin-derived molecule that was identified through fluorescence polarization (FP) assay-based high throughput screening (HTS) of a library of 6,000 compounds developed by the Kansas University Chemical Methodologies and Library Development (CMLD) center (www.cmld.ku.edu)^31^. The coumarin and dihydrocouramin-scaffold containing compounds (such as CMLD-2 and ita analogues) were developed by Tunge and co-workers using TFA-mediated hydroarylation chemistry and added to the in-house CMLD library for subsequent screening against various therapeutic targets^32^. CMLD-2 was demonstrated to efficiently disrupt the interaction between HuR protein and adenine and uridine-rich elements (ARE) oligo from Musashi RNA binding protein 1 (Msi1) at nanomolar concentrations^31^. Musashi is a known target of HuR^33^. CMLD-2 exhibited enhanced cytotoxicity towards HCT-116 colon and MiaPaCa2 pancreatic cancer cells and had an IC50 of 28.9 µM and 18.2 µM respectively. Accompanied with the cytotoxicity in HCT-116 cells was the reduction in mRNA and protein expression of Bcl-2, Msi1 and XIAP with concomitant increase in capase-3 and PARP cleavage and LC3I/II conversion indicating involvement of both autophagy and apoptosis-mediated death. In contrast, CMLD-2 exhibited reduced cytotoxicity towards normal fibroblast (WI-38; IC50 63.7 µM) and normal human colon epithelial (CCD 841 CoN IC50 63.7 µM) cells. The IC50 values for normal cells were approximately two-fold higher than for cancer cells. While HuR-targeted small molecule inhibitors such as CMLD-2 have been developed and tested in pancreatic and colon cancers, their efficacy against human lung cancer cells is not known.

In the present study, we examined whether pharmacologic inhibition of HuR using the small-molecule inhibitor, CMLD-2, would produce antitumor activity in human lung cancer cells. Demonstrating efficacy would provide a basis for making improvements in CMLD-2 -based cancer therapy.

## Materials and Methods

### Chemistry

CMLD-2 was prepared as previously reported^31^. The compound was dissolved in DMSO and used in the studies described herein. DMSO without the compound was used as drug carrier control in all of the studies.

### Cell lines and cell culture

Human non-small cell lung cancer (NSCLC) cells H1299, A549, HCC827, and H1975 and normal human lung fibroblasts MRC-9 and CCD16 were purchased from the American Type Culture Collection (ATCC, Manassas, VA). Cell lines were authenticated *via* STR profiling prior to initiating experiments. Tumor cells were maintained in RPMI 1640 supplemented with 10% fetal bovine serum (FBS; Sigma Aldrich, St. Louis, MO) and 1% penicillin/streptomycin. Normal human lung fibroblasts were cultured in EMEM with 10% fetal bovine serum (FBS; Sigma) and 1% penicillin/streptomycin.

### Cell viability assay

Cells (1 × 10^5^) were seeded in six-well plates in the appropriate culture medium containing 10% FBS. After 24 h of incubation, medium was replaced with fresh culture medium containing DMSO (drug carrier) or CMLD-2 (20 or 30 µM). At 24 h and 48 h after treatment, cells were harvested and cell viability was determined using trypan blue exclusion assay as previously described^20, 26^. The inhibitory activity of CMLD-2 was tested in duplicate well for each cell line and the experiment repeated three separate times. The data shown is representative of one experiment.

### Western blotting

Total cell lysates prepared from DMSO- and CMLD-2-treated cells were subjected to western blot analysis as previously described^20, 34, 35^. Primary antibodies against human HuR, Bcl2, Cyclin E, and p27 (Santa Cruz Biotechnology, Dallas, TX); BAX, Bcl-XL, caspase-3, caspase-9, and PARP (Cell Signaling, Cambridge, MA); and beta-actin (Sigma Chemicals) were purchased and used as recommended by the manufacturer. Appropriate horseradish peroxidase- (HRP)-tagged secondary antibodies (Santa Cruz Biotechnology, Inc., and Jackson Immuno-Research Laboratories, Inc., West Grove, PA) was used. Proteins were detected using an enhanced chemiluminescence kit (Thermo Scientific) on a chemiluminescence imaging system (Syngene, Frederick, MD) and the relative protein expression compared to beta-actin was quantified using Gene tools software (Syngene), as previously described^20, 36^.

### Quantitative real-time polymerase chain reaction (qRT-PCR)

qRT-PCR assay was performed as previously described^26, 27, 36^. Briefly, H1299 cells were collected and total RNA from DMSO- and CMLD-2-treated cells was isolated using TRIZOL (Invitrogen, Grand Island NY) reagent according to the manufacturer’s protocol. From the 2 µg of total RNA, first-strand complementary (c) DNA was synthesized with a Quant script cDNA synthesis kit (Bio-Rad, Richmond CA). The cDNA was subsequently used to perform qRT-PCR (Bio-Rad CFX96 Touch Real-Time PCR Detection System) with SYBR chemistry using iQTM SYBR Green super mix (Bio-Rad). The oligonucleotide primers specific for HuR have been previously described^26, 27^. The relative gene expression values were quantified as previously described^26, 27^. Experiment was conducted two separate times and the data obtained was analyzed for statistical significance. Data shown is from one of the two experiments.

### Cell cycle analysis

Cells (H1299, A549, MRC-9, CCD16; 1 × 10^*5*^/well) were treated with CMLD-2 for 24 h and 48 h following which they were harvested, stained with propidium iodide, and subjected to flow-cytometric analysis as described previously^20, 37, 38^. Cells treated with rapamycin (100 nM) served as positive control^39^. Cells treated with DMSO alone served as controls.

### Annexin V assay

H1299, A549, MRC-9 and CCD16 cells (1 × 10^5^/well) seeded in six-well plates were treated with CMLD-2 (30 µM) and stained with annexin V conjugated to fluorescein isothiocyanate (FITC) and propidium iodide (PI) using a Dead cell apoptosis kit (Molecular probes) according to manufacturer’s protocol. Cells treated with cisplatin (30 µM) served as positive control for the assay. Briefly, cells were harvested at 24 h and 48 h after treatment and suspended in annexin V binding buffer at a concentration of 1 × 10^5^ cells/ml. 100 µl of the cell suspension was incubated with 5 µl of annexin V FITC and 1 µl of 100 µg/ml PI. After 15 min of incubation at room temperature, the number of viable (annexin V- and PI-negative), early apoptotic (annexin V-positive and PI-negative), and dead (annexin V- and PI-positive) cells were determined with a FACSCalibur flow cytometer using the Cell Quest software (BD Biosciences) at excitation 488 nm and emission 530 nm. Results were plotted as the percentage of cells undergoing apoptosis at the two time points tested and subjected to statistical analysis. The apoptotic activity of CMLD-2 against each cell line was tested two separate times for reproducibility and statistical analysis. The data shown is representative of one experiment.

### Mitochondrial perturbation assay

H1299, A549, and MRC-9 cells (5 × 10^4^ cells/ well) seeded in chamber slides were treated with DMSO and CMLD-2 (30 µM). Cells treated with valinomycin (30 µM; Cayman, Ann Arbor, MI) served as positive control^40^. After 24 h and 48 h of treatment, cells were stained using the cationic dye JC-1 (Sigma Aldrich) as described previously^27, 34^. Briefly, the cells were incubated with JC-1 staining solution for 20 min at 37 °C. At the end of the incubation period, the staining solution was aspirated and cells were washed twice with culture medium. Cells were subsequently overlaid with fresh culture medium and observed under an inverted Leica SP2 MP confocal microscope (Leica Microsystems, Buffalo Grove, IL). Based on the changes in the membrane potential, the presence of JC-1 aggregates and JC-1 monomers was determined by excitation/emission at 525 nm/590 nm and 490 nm/530 nm, respectively. The quantitative difference in membrane potential was measured by determining the ratio of green over red fluorescence intensity and analyzed for statistical significance.

### Statistical analysis

All of the experiments were conducted two or more times and the data obtained for each of the study was subjected to statistical analysis. The SAS 9.2 software was used for statistical analyses. The results are presented as mean ± standard deviation (*SD*). Univariate statistical significance was determined by one-way analysis of variance (ANOVA) with Tukey’s adjustment for pairwise comparisons. Differences between groups were obtained using a linear mixed effects model with Tukey’s adjustment. A *P* value of less than 0.05 was considered statistically significant.

### Data availability statement

All data generated or analyzed during this study are included in this published article (and its Supplementary Information files).

## Results

### CMLD-2 preferentially inhibits lung tumor growth

**Figure 1:**
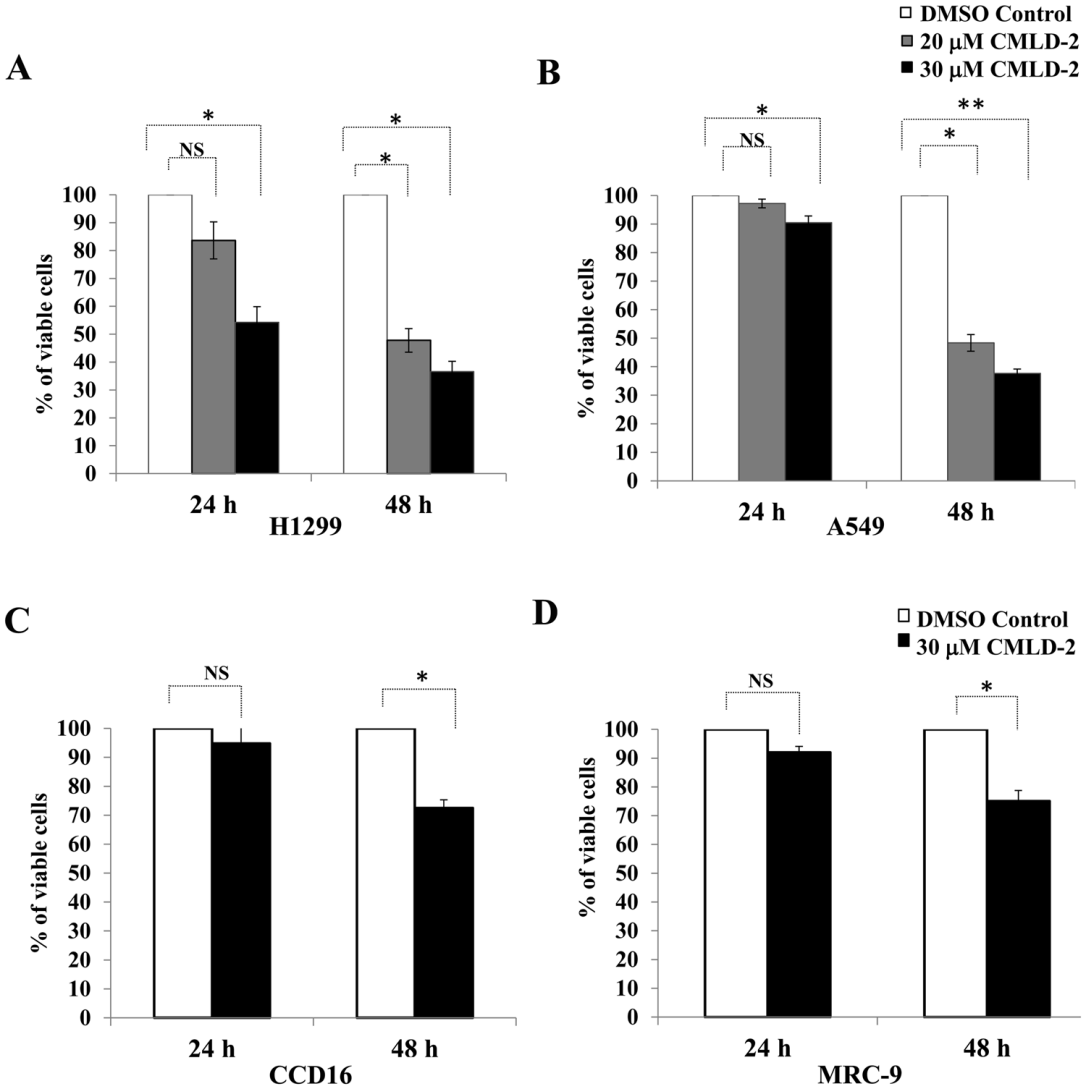
Human lung tumor (H1299, A549) and normal lung fibroblast (CCD16, MRC-9) cell lines were treated with either DMSO or CMLD-2 (20 or 30 µM). Cytotoxicity was measured at 24 h and 48 h after treatment. Error bar denotes *SD*; NS not significant; *p < 0.05; **p < 0.001.

Initial studies were focused on testing the cytotoxic concentration of CMLD-2 that was effective in suppressing tumor cell growth. A panel of NSCLC cells (H1299, A549, HCC827, H1975) was treated with 20 and 30 µM concentrations of CMLD-2 for 24 h and 48 h. A dose-dependent inhibition in cell viability was observed in all four CMLD-2 -treated cell lines compared with DMSO-treated control cells [Fig. 1A,B; *p* < 0.05; Supplementary Figure S1A,B; *p* < 0.05]. Treatment with 30 µM of CMLD-2, however, produced the greatest inhibition at the two time points tested. Based on this result, all subsequent studies were conducted with 30 µM CMLD-2.

Both the normal lung fibroblast cell lines (CCD16 and MRC-9) were less responsive and showed markedly less growth inhibition after CMLD-2 treatment (25–28% inhibition) than the tumor cells (45–67%; Fig. 1C,D).

### CMLD-2 treatment reduces expression of HuR and HuR-regulated mRNAs and proteins

**Figure 2:**
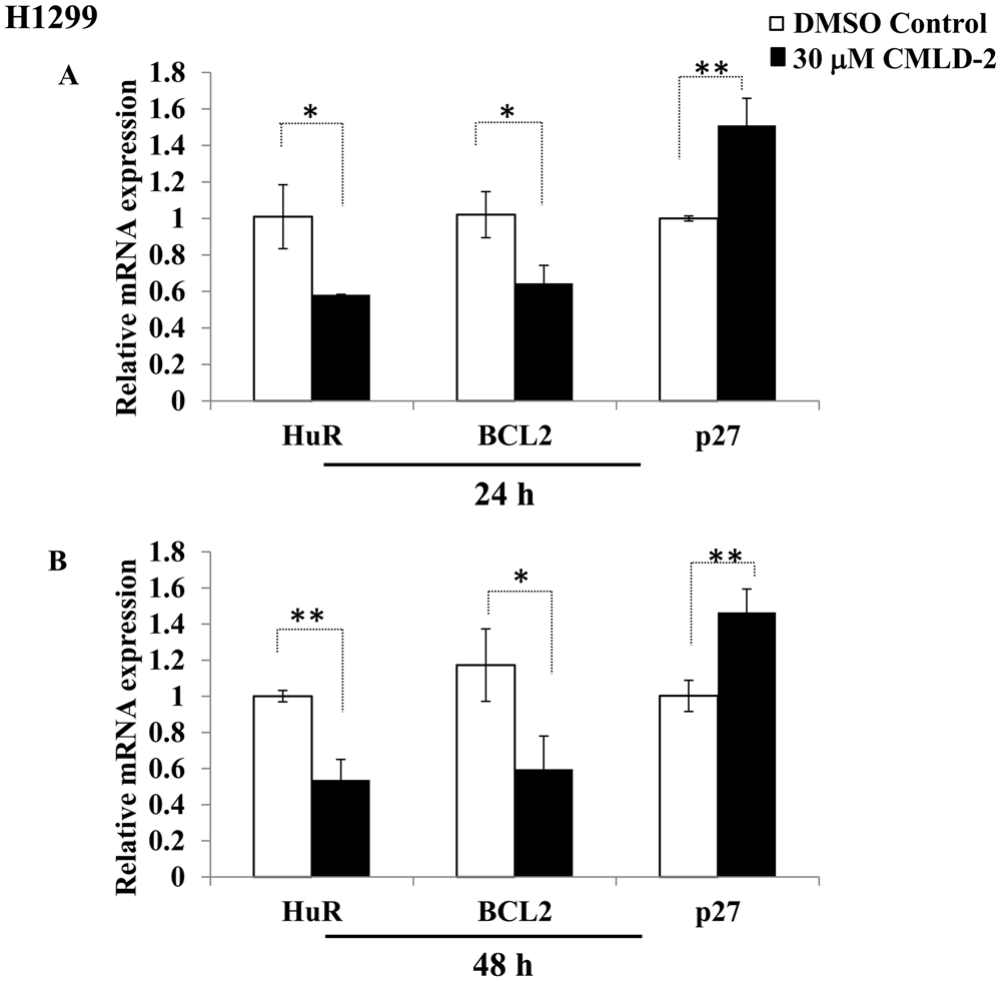
H1299 cells were treated with either DMSO or 30 μM CMLD-2 and the mRNA levels of HuR and HuR-regulated markers Bcl-2 and p27 were measured by RT-PCR. Error bar denotes *SD*; *p < 0.05; **p < 0.001.

**Figure 3:**
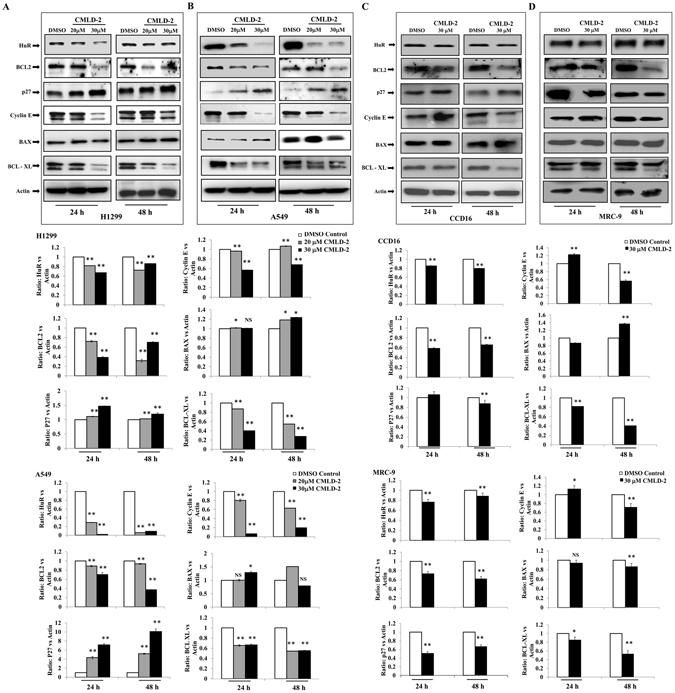
Expression of HuR and HuR-regulated proteins in lung tumor (H1299, A549) and normal lung fibroblast (CCD16, MRC-9) cells treated with either DMSO or 20 or 30 µM of CMLD-2. Bar graphs represent semi-quantitative analysis of the protein expression detected by western blotting. Beta-actin was used as internal loading control. Error bar denotes *SD*; NS not significant; *p < 0.05; **p < 0.001.

It has been previously shown that genetic knockdown of HuR results in attenuation of HuR mRNA and protein expression^14, 20, 27, 41–44^. To determine whether CMLD-2 treatment produced a similar reduction in HuR mRNA and protein expression, H1299 cells were treated with 30 μM CMLD-2 for 24 h and 48 h. A significant reduction in HuR and Bcl-2 mRNA expression, with a concomitant increase in p27 mRNA expression, was observed (Fig. 2; *p* < 0.05). Diminished protein expression of HuR, Bcl-2, Cyclin E and Bcl-XL, and increased expression of p27 and BAX, were associated with HuR mRNA reduction (Fig. 3A; *p* < 0.05). A similar change in the expression of these proteins was observed in CMLD-2-treated A549 (Fig. 3B; *p* < 0.05), H1975, and HCC827 cells (Supplementary Figures S2 and S3; *p* < 0.05). In normal cells, although CMLD-2 treatment produced changes in the expression of HuR and HuR-regulated proteins, the reduction was relatively less than that observed for tumor cells (Fig. 3C,D).

### CMLD-2 induces G1 cell cycle arrest

**Figure 4:**
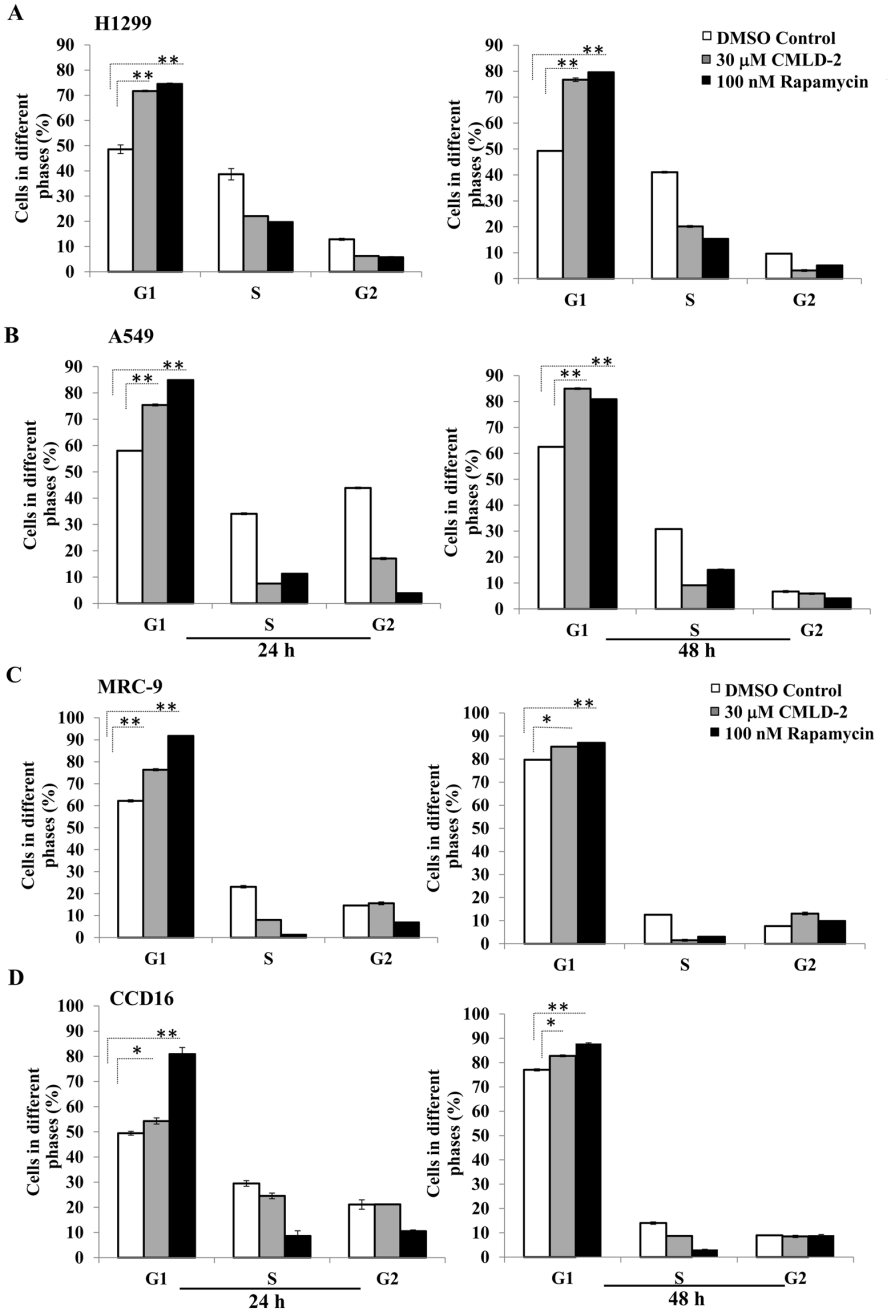
Cell cycle analysis showed that CMLD-2 induced greater G1 phase cell cycle arrest in H1299 and A549 cells than in MRC-9 and CCD16 cells at 24 h and 48 h after treatment. Rapamycin-treated cells served as positive control. Error bar denotes *SD*; NS not significant; *p < 0.05; **p < 0.001.

Since CMLD-2 treatment altered the expression of cell cycle proteins, such as p27 and cyclin E, we evaluated cell cycle profiles in tumor and normal cells. As shown in Fig. 4, a marked increase in the G1 phase of cell cycle was observed in both tumor and normal cells. However, the increase was greater in tumor cells than in normal cells at the two time points tested. In CMLD-2 -treated H1299 cells, an increase of 23% and 27% was observed at 24 h and 48 h, compared with DMSO-treated control cells. In CMLD-2 -treated A549 cells, the increase was 17% and 22% over DMSO-treated control cells. In CMLD-2 -treated MRC-9 cells, 14% and 6% increases over controls were observed. The increase in G1 phase of cell cycle in CCD16 cells was only 5% at the two time-points tested. In all cell lines, rapamycin treatment used as positive control showed maximum G1 cell cycle arrest.

### CMLD-2 induces mitochondrial perturbation in tumor cells

**Figure 5:**
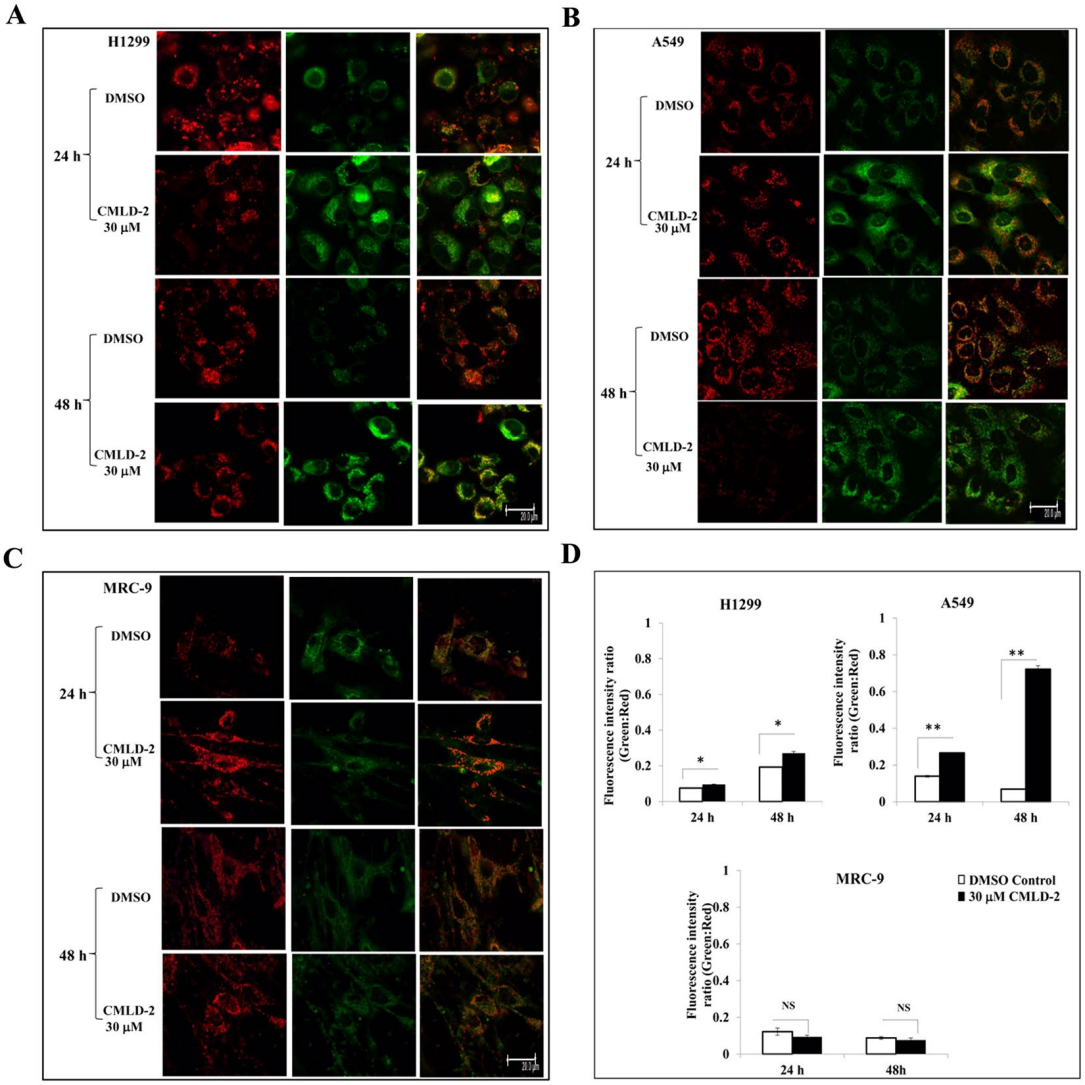
CMLD-2 perturbs the mitochondrial membrane potential in cells. Perturbation of mitochondrial membrane potential using JC-1 dye was observed to be greater in CMLD-2 -treated tumor (H1299 and A549) cells, compared to normal (MRC-9) cells. MRC-9 cells with high membrane potential show an aggregation of fluorescent red dye, whereas the H1299 and A549 cells with low potential display enhanced monomeric green fluorescence. Red fluorescence shows aggregation of fluorescent red dye indicating intact mitochondrial potential and monomeric green fluorescence indicates perturbation and low mitochondrial potential. Overlay image shows the degree of perturbation. Bar graph shows the relative fluorescence intensity in DMSO- and CMLD-2-treated cells. Error bar denotes SD; NS not significant; *p < 0.05; **p < 0.001. Scale bar, 200 µM.

Studies have shown that treatment of cells with cytotoxic agents induces cell death by perturbing the mitochondria^34, 45–47^. In the present study, we observed an increase in pro-apoptotic Bax and a decrease in anti-apoptotic Bcl-XL and Bcl-2 protein expression (Fig. 3). All of these proteins are known to be localized in the outer mitochondrial membrane; any perturbation in the mitochondria results in changes in the expression of these proteins. We therefore investigated whether CMLD-2 treatment produced alterations in the mitochondria. As shown in Fig. 5, a remarkable and significant increase in mitochondrial perturbation was observed in CMLD-2 -treated H1299 and A549 tumor cells, compared to CMLD-2 -treated MRC-9 cells, at both 24 h and 48 h after treatment (*p* < 0.05). Increased mitochondrial perturbation was also observed in valinomycin-treated tumor cells (Supplementary Figure S4; *p* < 0.05). Valinomycin is a known inducer of mitochondrial perturbation^40^. That the low mitochondrial potential observed in CMLD-2-treated MRC-9 cells was due to inability of the cells to respond to treatment was eliminated by the observed increase in perturbation in valinomycin-treated cells (Supplementary Figure S4; *p* < 0.05). These results clearly demonstrate that tumor cells are more susceptible to mitochondrial perturbation when exposed to CMLD-2 than are normal cells.

### CMLD-2 treatment activates caspases and induces apoptotic cell death

**Figure 6:**
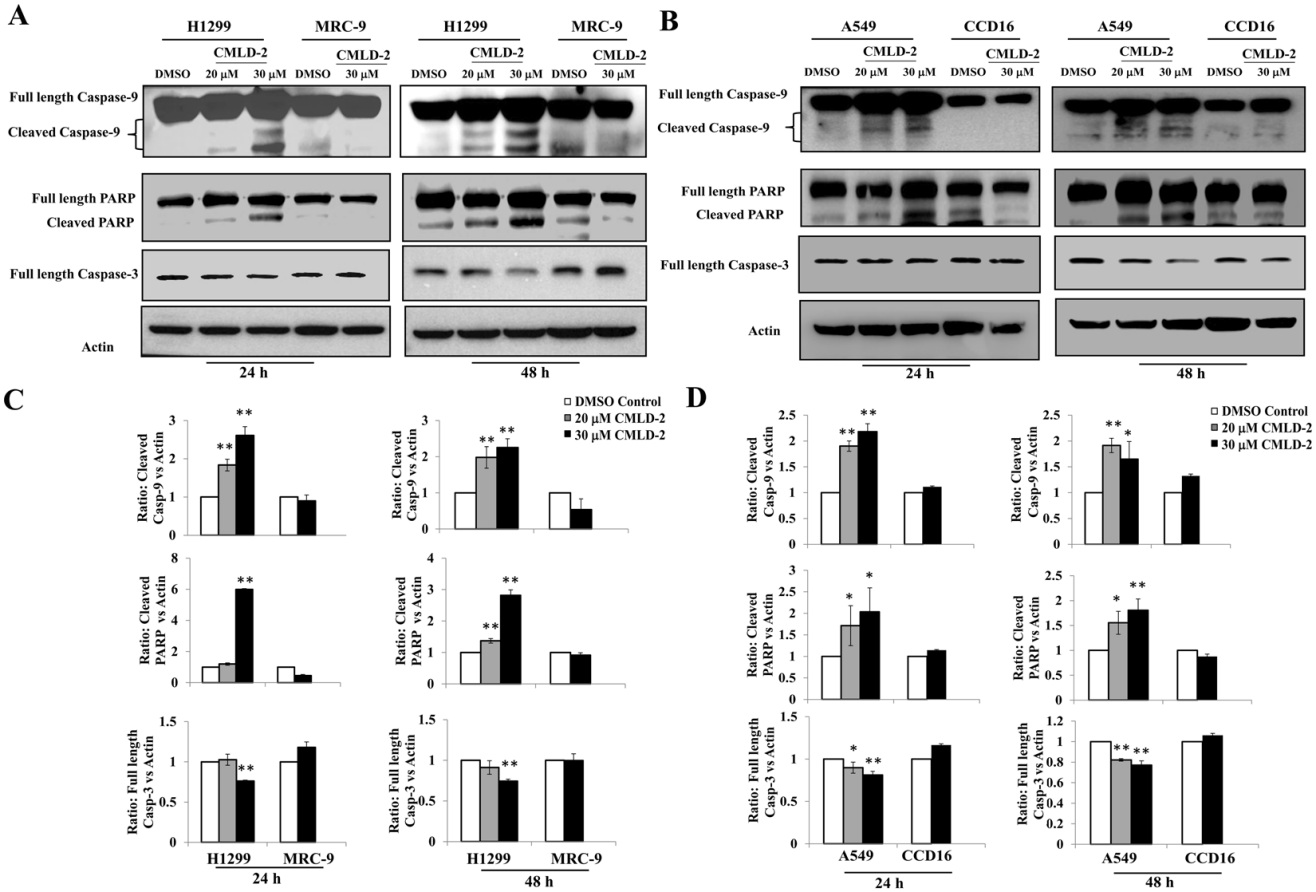
CMLD-2-induced apoptosis was observed in H1299 and A549 cells, but not in MRC-9 cells and CCD16. Induction of apoptosis was measured by cleavage of caspase-3, caspase-9, and PARP at 24 and 48 h and CCD16 after treatment, compared with DMSO-treated cells. Bar graphs represent semi-quantitative analysis of the protein expression detected by western blotting. Beta-actin was used as internal loading control. Error bar denotes *SD*; NS not significant; *p < 0.05; **p < 0.001.

**Figure 7:**
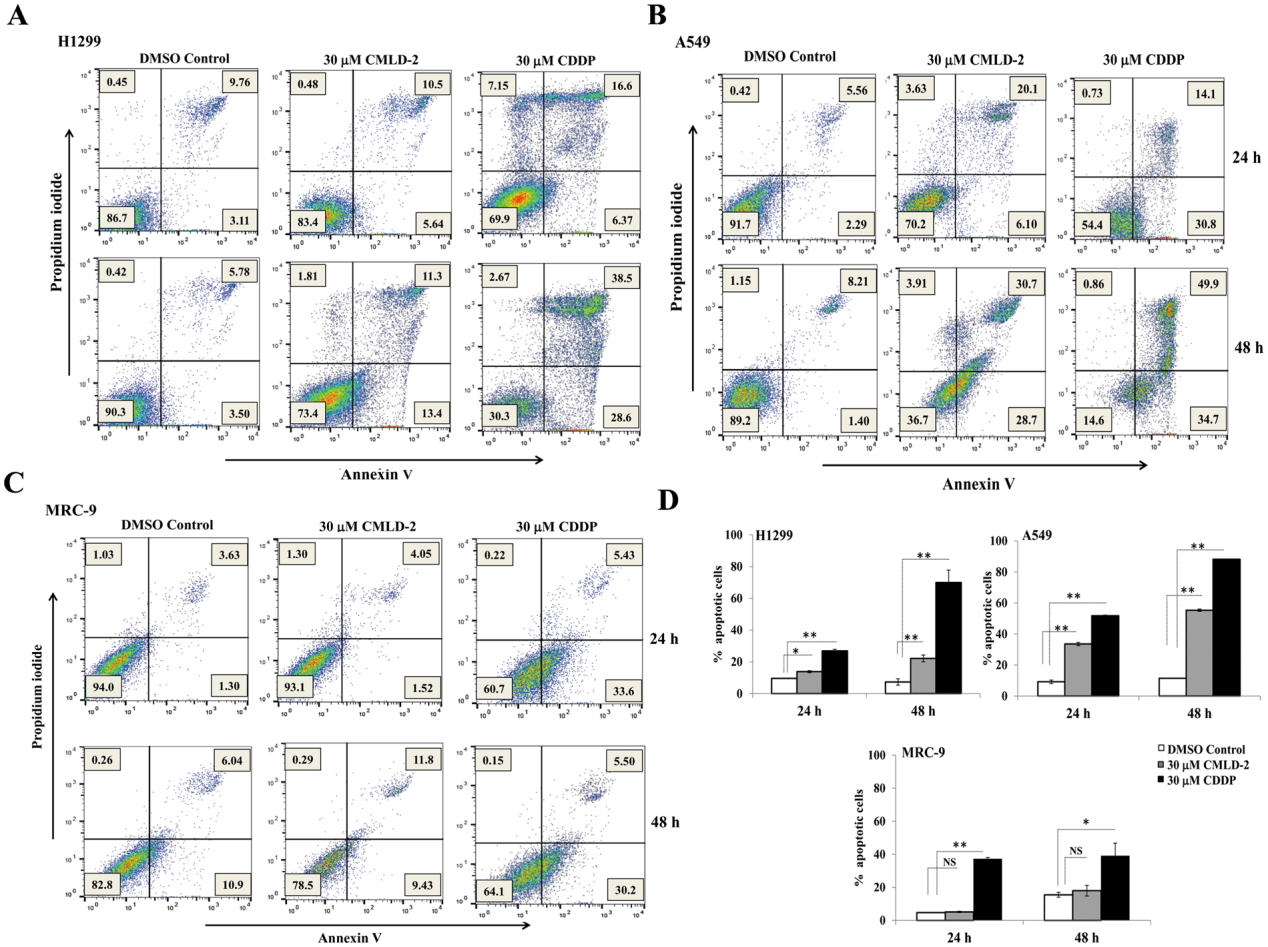
Annexin V staining demonstrate CMLD-2 treatment induces apoptosis. Flow cytometric analysis shows the percentage of apoptotic and necrotic cells (Q1: necrotic; Q2: late apoptotic; Q3: early apoptotic; Q4: Live cells) at 24 h and 48 h in CMLD-2 -treated cells. (**A**) H1299; (**B**) A549; (**C**) MRC-9. Induction of apoptosis was greater in CMLD-2 -treated H1299 and A549 cells than in CMLD-2 -treated MRC-9 cells. (**D**) Bar graphs represent the percentage of apoptotic cells at 24 h and 48 h after CMLD-2 treatment. Cells treated with cisplatin (CDDP) served as positive control for each cell line. Error bar denotes *SD*; NS not significant; *p < 0.05; **p < 0.001.

To establish whether the increased expression of pro-apoptotic BAX protein and simultaneous reduction of anti-apoptotic proteins Bcl2 and Bcl-XL in CMLD-2-treated tumor cells occurred through apoptosis, we performed western blot analysis for apoptotic proteins. Marked activation of caspase-9 and -3 was observed in CMLD-2 -treated H1299 and A549 cells, resulting in cleavage of their substrate, PARP (Fig. 6A,B; *p* < 0.05). Similar observations were made in H1975 and HCC827 cells (Supplementary Figure S5; *p* < 0.05). Correlating with caspase activation was the significant increase in annexin-V-positive staining in CMLD-2 -treated H1299 and A549 cells (Fig. 7A,B,D; *p* < 0.05). In normal (MRC-9 and CCD16) cells, no marked activation of caspase-3, -9 or PARP cleavage was seen after CMLD-2 treatment at either time point (Fig. 6A,B). Analysis for annexin-V-positive staining in CMLD-2 treated MRC-9 cells showed no significant change compared with DMSO-treated control cells (Fig. 7C,D). These results demonstrate CMLD-2 treatment increases apoptotic cell death in tumor cells compared to normal cells.

## Discussion

HuR, an RNA-binding protein, has been demonstrated to play a role in tumor growth, progression, and metastasis^1–14, 48^. Studies have shown that HuR overexpression in human cancers not only correlates with poor prognosis, but also with resistance to therapy, thus establishing HuR as a molecular target for therapy^11–17^. Earlier studies from our laboratory and others have shown that siRNA-/shRNA-based genetic silencing of HuR in a variety of cancer cells resulted in inhibition of cell proliferation, reduced cell migration and invasion, and suppression of metastasis^7, 18–22^. While these studies have established the proof-of-concept, advancing HuR siRNA/shRNA gene-based cancer therapy has limitations^23, 24^. An alternate approach is pharmacologic inhibition, which can have sustained inhibitory activity and effective anticancer activity. Therefore, in the present study, we tested the efficacy of a small-molecule HuR inhibitor, CMLD-2, against human lung cancer cells compared with normal lung fibroblasts.

Our initial studies focused on determining the effective drug concentration, and identified that 30 µM CMLD-2 produced the maximum inhibitory effect on human lung cancer cells. Using this concentration in subsequent studies, CMLD-2 exhibited preferential cytotoxicity towards non-small cell lung cancer cells (H1299, A549, H1975, and HCC827) compared to the normal human fibroblasts (MRC-9 and CCD16). CMLD-2-mediated inhibition was accompanied by a G1 phase cell cycle arrest that subsequently led to apoptosis. Molecular studies demonstrated that CMLD-2 treatment significantly reduced HuR mRNA and protein expression and HuR-regulated oncogenic proteins in tumor cells compared with normal cells. While our results clearly and convincingly demonstrate that CMLD-2 is effective against lung cancer cells, the question of how CMLD-2 targets HuR and exhibits selectivity to cancer cells compared to normal cells, when HuR is expressed in both cell types, is unclear. Although, Wu *et al*.^31^, showed that CMLD-2 exerts its antitumor activity by disrupting the interaction between HuR protein and its target mRNAs, the binding affinity to HuR in tumor cells versus normal cells have not been studied. While in the present study we have not investigated the mechanism for tumor selectivity, we believe that the binding of CMLD-2 to HuR is likely greater in tumor cells, since they express relatively higher levels of HuR than do normal cells. Further, tumor cells may be addicted to the survival signals provided by HuR-regulated oncoproteins. Thus, CMLD-2-mediated suppression of HuR results in reduced expression of HuR-regulated oncoproteins, leading to enhanced tumor cell killing compared to normal cells.

In a recent study, we showed the siRNA-mediated HuR inhibition combined with radiation produced radiosensitization of breast cancer cells^27^. In that study, we demonstrated that radiosensitization involved mitochondrial perturbation and free-radical production that was accompanied by DNA damage. Similar to our previous observations, CMLD-2 treatment also resulted in mitochondrial perturbation that was significantly higher in H1299 and A549 tumor cells than in normal MRC-9 cells. Our results show that although CMLD-2 and HuR specific siRNA operate at two different levels upstream to inhibit HuR, they appear to converge on common death signaling pathways downstream resulting in tumor cell killing. While our study indicates apoptotic cell death, the report by Wu *et al*.^31^, described CMLD-2 treatment induced both autophagy and apoptosis in colon and pancreatic cancer cells. Thus, it is will be of interest to investigate whether autophagy occurs in CMLD-2-treated lung cancer cells also. Further, determining whether autophagy in CMLD-2 treated lung cells provide survival or death signal will not only increase our understanding of the modus operandi of CMLD-2 but likely offer an opportunity to combine CMLD-2 with autophagy inducers to produce enhanced anticancer activity.

The ability of CMLD-2 to exhibit profound antitumor activity supports further development of the inhibitor that is efficacious at lower drug concentrations in the nanomolar range and that will have clinical relevance. Our *in vitro* results, while promising, warrant testing the *in vivo* efficacy, pharmacokinetics and pharmacodynamics, and toxicity of CMLD-2. Results from *in vivo* studies will provide an opportunity to develop structure-activity relationship (SAR)-based CMLD-2 inhibitors and a strong rationale to *develop* HuR-targeted small molecule therapeutics for treating lung cancer and other solid tumors.

In conclusion, we have established proof-of-concept and shown that CMLD-2 represents a promising HuR-targeted therapeutic class for cancer treatment. However, further improvements in CMLD-2 and its analogues that exhibit efficacy at nanomolar concentrations are warranted. The availability of such HuR-targeted small molecule therapeutics that highly efficacious at clinically relevant doses will make a significant impact not only in lung cancer treatment but also in the treatment of other HuR overexpressing solid tumors.

## Electronic supplementary material

Supplementary Information
