# Supplementary material for: HuR-targeted small molecule inhibitor exhibits cytotoxicity towards human lung cancer cells
Source: Sci Rep. 2017 Aug 30;7:9694. doi: 10.1038/s41598-017-07787-4 (PMC5577245; doi:10.1038/s41598-017-07787-4)
Supplement: Supplementary file 1 — Supplementary Information [file 41598_2017_7787_MOESM1_ESM.pdf]

HuR-targeted small molecule inhibitor exhibits cytotoxicity towards human lung cancer cells

Ranganayaki Muralidharan[1,4], Meghna Mehta[2,4], Rebaz Ahmed[1,5], Sudeshna Roy[6], Liang Xu[7], Jeffrey Aubé[6], Allshine Chen[3], Yan Daniel Zhao[3,4], Terence Herman[2,4], Rajagopal Ramesh[1,4,5*], Anupama Munshi[2,4*]

Departments of [1]Pathology, [2]Radiation Oncology, [3]Epidemiology and Statistics, University of Oklahoma Health Sciences Center, Oklahoma City, Oklahoma 73104, USA; [4]Stephenson Cancer Center, University of Oklahoma Health Sciences Center, Oklahoma City, Oklahoma 73104, USA; [5]Graduate Program in Biomedical Sciences, University of Oklahoma Health Sciences Center, Oklahoma City, Oklahoma 73104, USA; [6]Division of Chemical Biology, University of North Carolina, Chapel Hill, North Carolina 27599, USA; Department of Molecular Biosciences, University of Kansas Medical Center, Kansas City, Kansas 66160, USA.

***Address for Correspondence:**

Anupama Munshi, Department of Radiation Oncology, University of Oklahoma Health Science Center, 975 N.E., 10th Street, Oklahoma City, OK 73104, USA; Phone: (405) 271-6102; Email: anupama-munshi@ouhsc.edu

Rajagopal Ramesh, Department of Pathology, University of Oklahoma Health Science Center, 975 N.E., 10th Street, Oklahoma City, OK 73104, USA; Phone: (405) 271-6101; Email: rajagopal-ramesh@ouhsc.edu

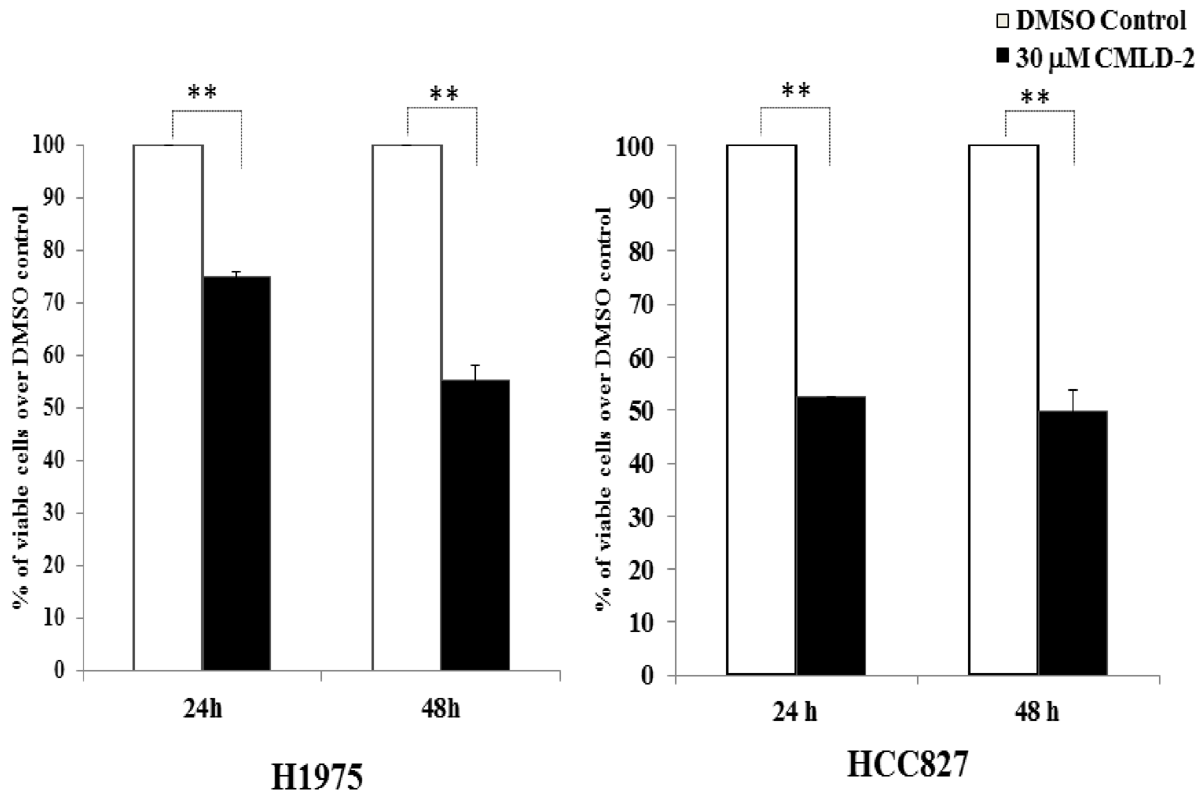

Figure S-1. H1975 and HCC827 lung tumor cells were treated with either DMSO or 30 µM of CMLD-2 and the cytotoxicity was measured at 24 h and 48 h after treatment. Error bar denotes *SD*; NS, not significant;*p<0.05; **p<0.001

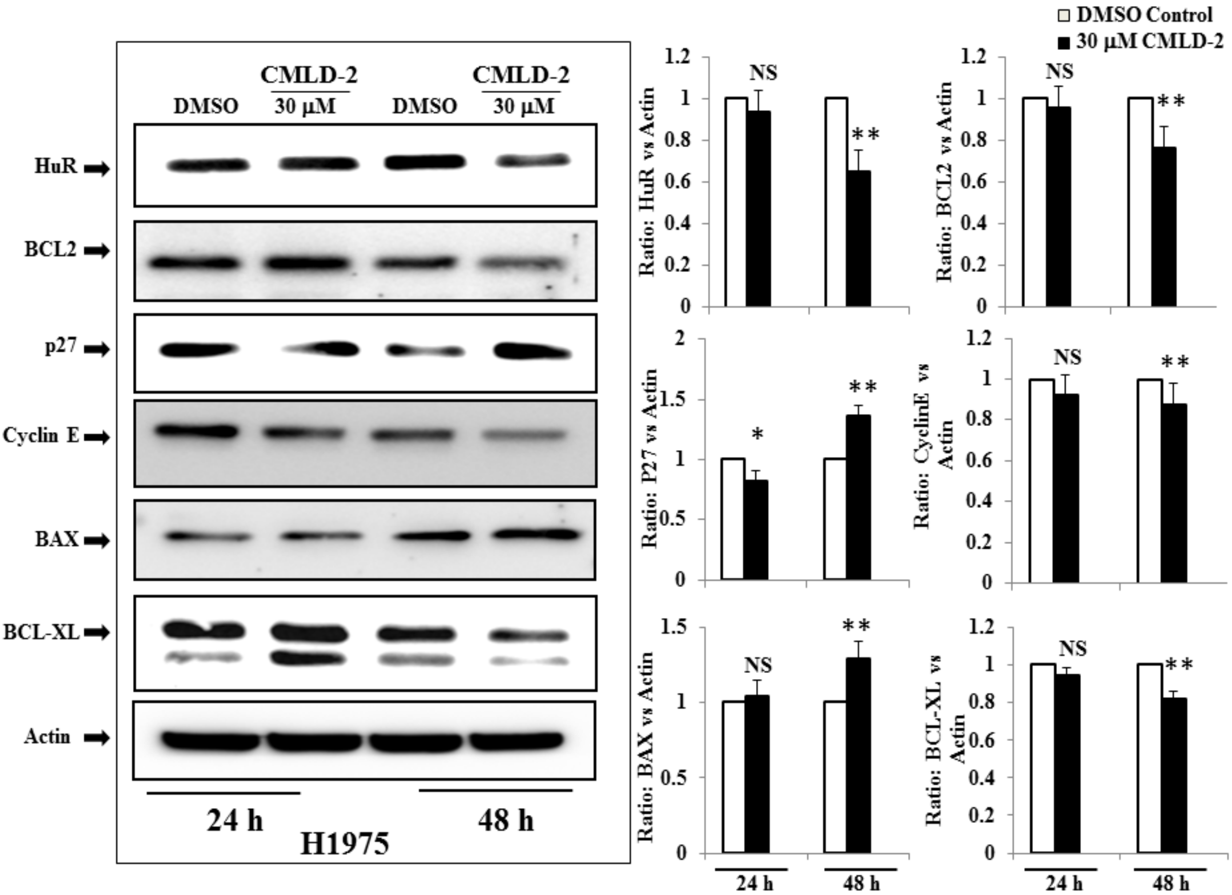

Figure S-2. Western blot analysis shows the expression of HuR and HuR regulated proteins in H1975 cells treated with DMSO or 30 μM of CMLD-2. Bar graphs represent semi-quantitative analysis of the protein expression. Error bar denotes *SD*; NS not significant;*$p < 0.05$; **$p <0.001$

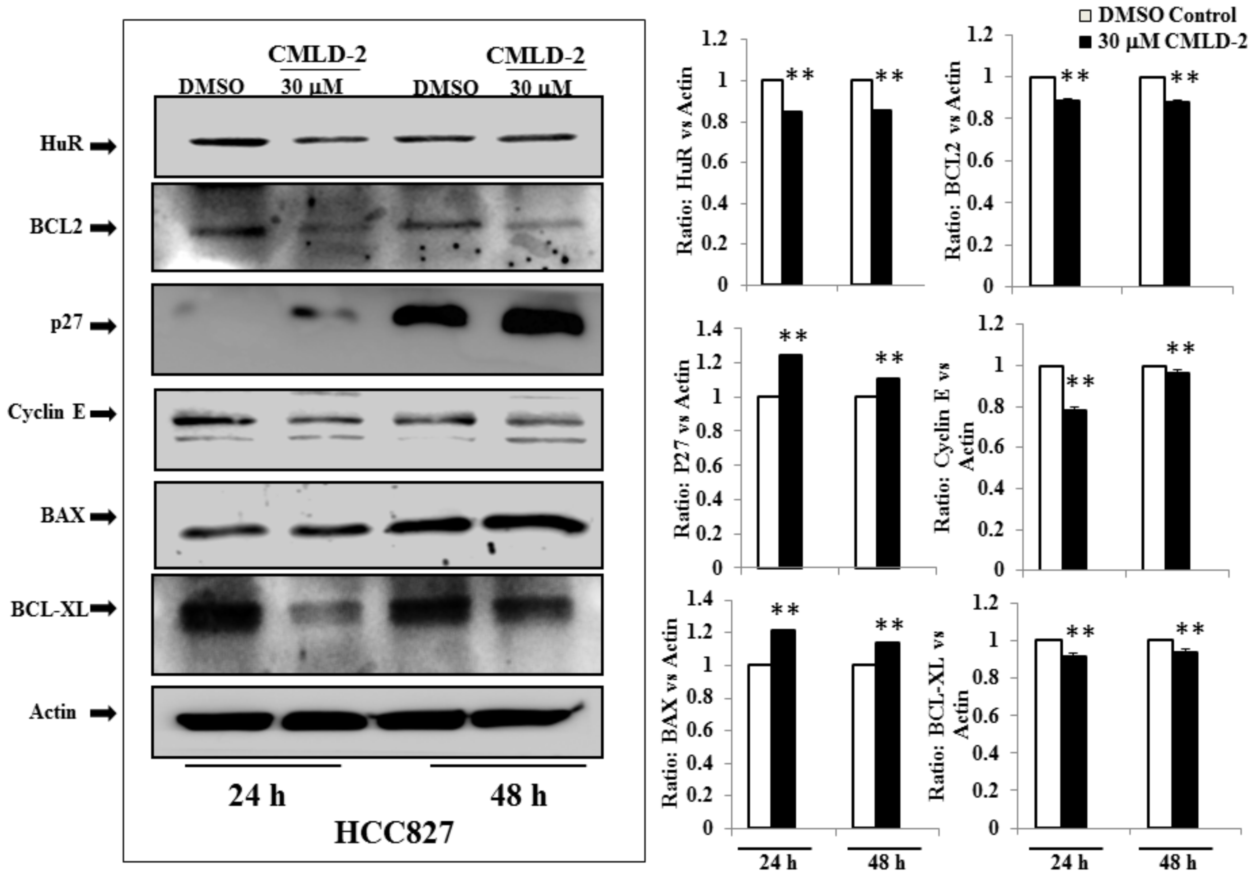

Figure S-3. Western blot analysis shows the expression of HuR and HuR regulated proteins in HCC827 cells treated with DMSO or 30 μM of CMLD-2. Bar graphs represent semi-quantitative analysis of the protein expression. Error bar denotes *SD*; NS not significant; **p*<0.05; ***p*<0.001

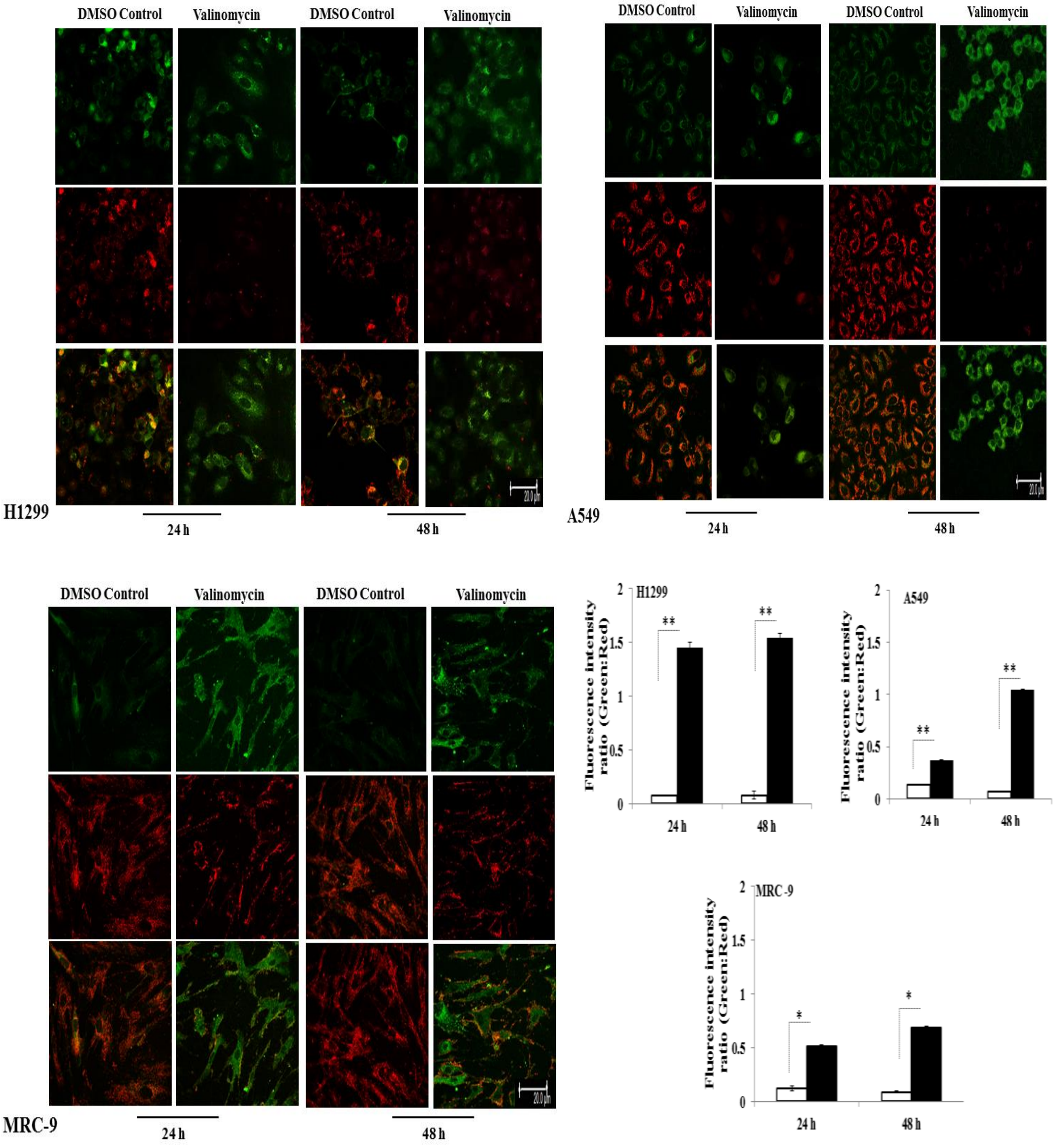

Figure S-4. Mitochondrial perturbation in tumor and normal cells treated with DMSO or Valinomycin (30 µM). Red fluorescence shows aggregation of fluorescent red dye indicating intact mitochondrial potential and monomeric green fluorescence indicates perturbation and low mitochondrial potential. Overlay image shows the degree of perturbation. Bar graphs represent the relative fluorescence intensity in DMSO- and Valinomycin-treated cells. Error bar denotes SD; $p<0.05$; ** $p< 0.001$

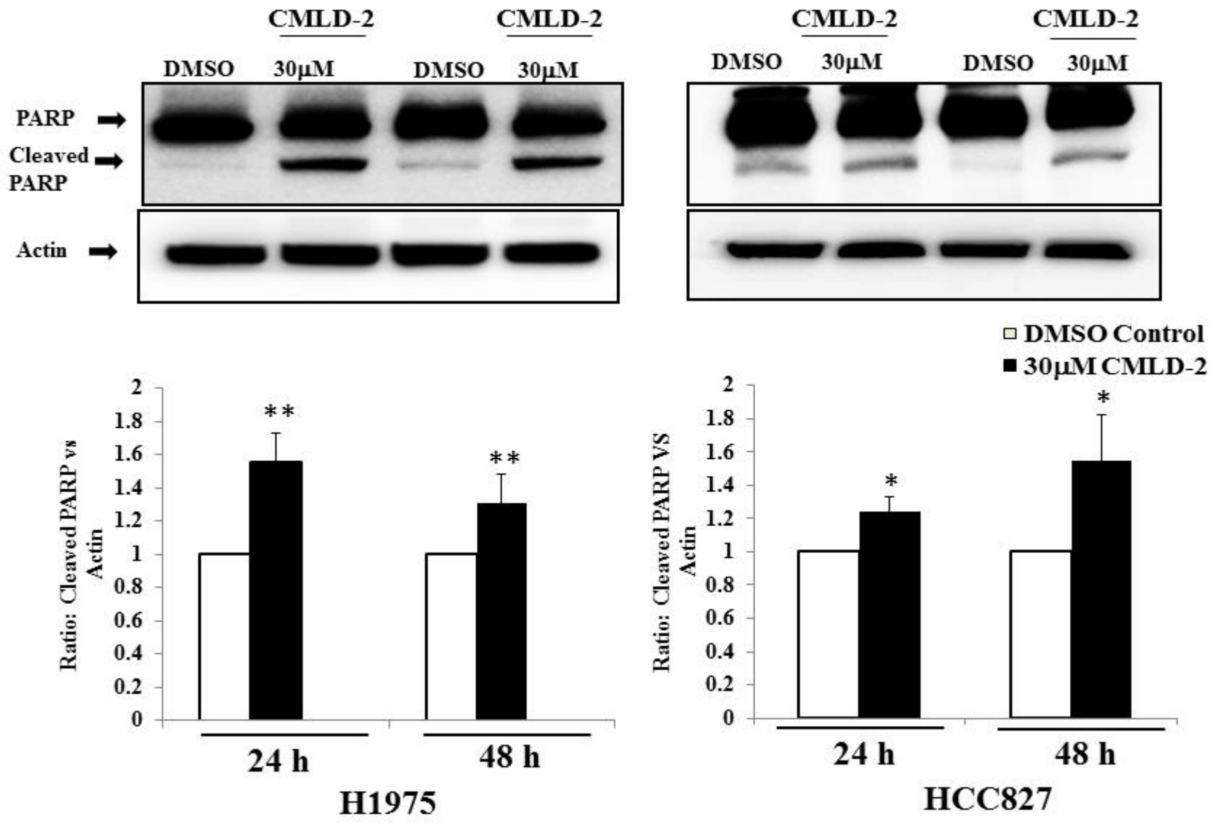

Figure S-5. Western blot analysis shows the expression of cleaved PARP in H1975 and HCC827 cells treated with either DMSO or 30 μM of CMLD-2. Bar graphs represent semi-quantitative analysis. Error bar denotes *SD*; NS, not significant;*p<0.05; ** p< 0.001
